# Preparation and characterization of a fully human monoclonal antibody specific for human tumor necrosis factor alpha

**DOI:** 10.1080/21655979.2021.1967710

**Published:** 2021-12-09

**Authors:** Xinmei Liao, Hui Liang, Jian Pan, Qian Zhang, Jiaqi Niu, Cuili Xue, Jian Ni, Daxiang Cui

**Affiliations:** a Institute of Nano Biomedicine and Engineering, Shanghai Engineering Research Centre for Intelligent Diagnosis and Treatment Instrument, Department of Instrument Science and Engineering, School of Electronic Information and Electrical Engineering, Shanghai Jiao Tong University, Shanghai, P. R. China; b National Center for Translational Medicine, Collaborative Innovational Center for System Biology, Shanghai Jiao Tong University, Shanghai, P. R. China; c National Engineering Research Center for Nanotechnology, Shanghai, P. R. China; d Nuance Biotech (Shenzhen) Co. Ltd, Shenzhen, Guangdong, P. R. China; e Institute of Pediatric Research, Children’s Hospital of Soochow University, Suzhou, Jiangsu, P. R. China

**Keywords:** Fully human monoclonal antibody, tumor necrosis factor α, rheumatoid arthritis, affinity, inhibitor

## Abstract

Tumor necrosis factor alpha (TNFα) is an important inflammatory factor. It plays a cardinal role in inflammatory synovitis and articular matrix degradation, and is, therefore, a prime target for directed immunotherapy in autoimmune diseases. In this study, we screened and isolated the B cells secreting anti-TNFα antibody from patients with rheumatoid arthritis. The heavy-chain and light-chain sequences of the antibody were cloned and used to generate a stable Chinese hamster ovary (CHO) cell line producing the antibody, which was named Haidalimumab. Haidalimumab showed a TNFα binding affinity comparable to that of the antibody Humira, which is the best TNF inhibitor on the market. Furthermore, Haidalimumab could effectively neutralize recombinant human tumor necrosis factor alpha (rhTNFα) toxicity in a C57BL/6 mouse model and showed significant therapeutic effect in a tumor necrosis factor transgenic (TNF-Tg) mouse arthritis model. In conclusion, we developed a high-affinity, fully human anti-TNFα antibody with low immunogenicity that could potentially have significant therapeutic applications in rheumatoid arthritis or other autoimmune diseases.

**Abbreviations:** ELISAenzyme linked immunosorbent assayRArheumatoid arthritisSDS-PAGEsodium dodecyl sulfate polyacrylamide gel electrophoresisrhTNFαrecombinant human tumor necrosis factor-alphaEC_50_concentration for 50% of maximal effectTNF-Tg micetumor necrosis factor transgenic miceAMDactinomycin DMTTmethylthiazolyldiphenyl-tetrazolium bromidePBSphosphate‐buffered saline

## Graphical abstract

**Figure:**
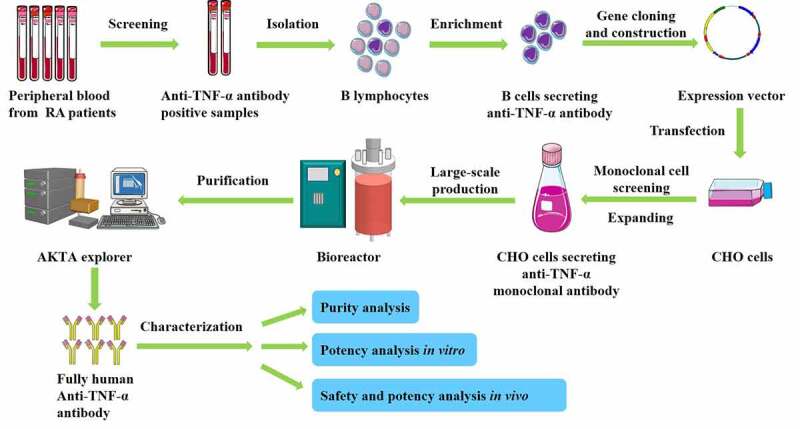

## Introduction

The incidence of autoimmune diseases such as rheumatoid arthritis (RA) is high. The World Health Organization lists rheumatism, cardiovascular disease, and cancer as the top three threats to human health. A dysregulated inflammatory process characterizes most autoimmune diseases [1], and tumor necrosis factor alpha (TNFα) is an important inflammatory factor [2]. TNFα levels are high in tumor-infiltrating dendritic cells and inflammatory cells [3]. It is overproduced in rheumatoid joints primarily by macrophages. TNFα induces the production of other proinflammatory cytokines such as interleukin (IL)-1 and IL-6, increases endothelial layer permeability and the expression of adhesion molecules, activates neutrophils and eosinophils, and induces acute phase reactants and tissue-degrading enzymes production in synoviocytes and chondrocytes. Overexpression of TNFα is associated with autoimmune diseases such as RA, psoriasis, lupus erythematosus, ankylosing spondylitis, and multiple sclerosis, among other effects. Although the causes of RA are not fully understood, TNFα seems to play a key role in a variety of events in inflammatory synovitis and articular matrix degradation, and is, therefore, a primary target for directed immunotherapy in RA. Antibodies and soluble TNF receptors that bind TNFα with high specificity neutralize its activity and have been developed for use as therapeutic agents.

Five TNF inhibitors have been approved by the U.S. Food and Drug Administration for RA treatment: Remicade (Infliximab, 1998), Enbrel (Etanercept, 2001) [4–9], Humira (Adalimumab, 2003) [10–14], Simponi (Golimumab, 2011) [15–18], and Cimzia (Certolizumab Pegol, 2010) [19,20]. Remicade is a chimeric monoclonal antibody that contains sequences of murine origin and has immunogenicity. Enbrel is a human recombinant receptor/Fc fusion protein. Humira is a human monoclonal antibody which was developed by phage display technology from a human-sourced phage display antibody library. Simponi is a humanized antibody derived from TNF-immunized transgenic mice engineered to express human IgG. Cimzia is a recombinant, humanized F(ab)’ fragment of an antibody conjugated to polyethylene glycol to enhance its plasma half-life (Table 1). So far, no human-derived TNF antibody has been made available on the market. The marketed anti-TNFα therapeutic agents for RA treatment have limitations concerning the affinity, stability, solubility, and the immunogenicity of the antibodies used [21–23]. Notably, they may be limited by the immune responses to their non-human elements or artificially fused sequences.

**Table 1.** A summary of the characteristics of FDA-approved TNF inhibitors and Haidalimumab

Characteristics	Infliximab	Adalimumab	Golimumab	Certolizumab	Etanercept	Haidalimumab
Origin	Chimeric, murine Fab	Fully human, recombinant human Fab	Fully human	Humanized Fab	TNF receptor fusion protein	Fully human
Biologic type	IgG1 kappa	IgG1 kappa	IgG1 kappa	Fab of IgG4	Fc of IgG1	Fc of IgG1
Molecular weight (kDa)	150	150	150	91	150	150
Specificity	TNF-α	TNF-α	TNF-α	TNF-α	TNF-α / LT-α	TNF-α
TNF ligands	sTNF, tmTNF	sTNF, tmTNF	sTNF, tmTNF	sTNF, tmTNF	sTNF, tmTNF,	sTNF, tmTNF,
Mode of production	Culture in CHO cells	Fab produced by phage display, culture in CHO cells	Produced in transgenic mice	Expressed in *Escherichiacoli* and conjugated to polyethylene glycol	DNA recombinant technology in CHO cells	DNA recombinant technology in CHO cells

TNF: tumor necrosis factor, sTNF: soluble TNF, tmTNF: transmembrane TNF, LT-α: lymphotoxin-α.

This study was aimed at researching and developing TNFα antibodies with low immunogenicity and with functions similar to that of human antibodies. We isolated and screened B cells secreting anti-TNFα antibody from rheumatoid arthritis patients. We cloned the heavy chain and the light chain sequences of anti-TNFα antibody and constructed a stable CHO cell line to produce the antibody Haidalimumab. We characterized the biological activity and the efficacy of Haidalimumab *in vitro* and *in vivo*. Subsequently, Haidalimumab was observed to have low immunogenicity and is postulated to possess higher therapeutic potential than the currently available TNF inhibitors.

## Materials and methods

### Isolation and cloning of anti-TNFα-positive B cells

This study was approved by the ethics committee of Shanghai Jiao Tong University. An indirect enzyme-linked immunosorbent assay (ELISA) was used to select the human B cells that secrete higher levels of anti-human TNFα antibody. A 96-well microplate was coated with 2 μg/mL human TNFα antigen (C008, Shanghai Sinobio, China), blocked with 5% nonfat dry milk at 25°C for 2 h, and was washed thrice. Patient plasma (100 μL) was added to each well and the plate was incubated at RT for 1 h and washed thrice. Sheep anti-human IgG-HRP was then added and the plate was incubated at RT for 1 hour and washed thrice. Subsequently, tetramethyl benzidine (TMB) solution was added and the plate was incubated at RT for 10 min. 1 N H_2_SO_4_ (50 μL) was then added to the solution to terminate the reaction. The OD_450_ values were read using a microplate reader and the blood samples with high OD values were selected for further analysis.

To isolate and enrich human B-cells secreting anti-TNFα antibody, the selected blood samples were diluted with PBS (phosphate‐buffered saline, pH 7.2, 50 mM) and superimposed on Ficoll-Paque PREMIUM (17–5442-02, General Electric Company, Boston, Massachusetts, USA). The separation tubes were then centrifuged at 1500 *× g* for 30 min at 4°C. The supernatants consisting mainly of peripheral blood mononucleocytes (PBMCs) were collected, transferred to fresh tubes, and washed thrice with PBS. A biopanning method was used to screen anti-TNFα-positive B cells from the obtained PBMC mixture, as follows: a 24-well plate was coated with 2 μg of TNFα antigen per well overnight and blocked with 5% nonfat dry milk at 25°C for 2 h. PBMCs (2 mL) were added to each well followed by incubation for 2 h at 37°C. B cells secreting anti-TNFα antibodies were thus, enriched and collected. To culture and screen human B-cells secreting anti-TNFα antibodies after enrichment and collection, macrophages and dendritic cells were used as feeding cells, which provide growth factors and hematopoietic factors, to maintain the proliferation and the differentiation of the B cells. The feeding cells, at an initial concentration of 3 × 10^5^ cells/mL, were maintained at 37°C (5% CO_2_) in Roswell Park Memorial Institute-1640 (RPMI-1640, Thermo Fisher Scientific, Waltham, MA, USA) supplemented with 10% (v/v) fetal bovine serum (Biological Industries, Cromwell, CT, USA), 100 U/mL penicillin, and 1 mg/mL streptomycin. When the feeding cells reached a level of 80%-90%, three to five B-cells were added to each well with feeding cells. Subsequently, 100 μL of Burkitt lymphoma virus from the cell supernatant of B958 cells (purchased from the Pathology and Physiology Research Office of Chongqing Medical University, China) was added. Half of the cell supernatant in the wells was replaced with fresh culture medium every 4 days. After 2 days in the culture, the B-cell colonies formed were observed using the Olympus BX41 imaging system (Olympus, Tokyo, Japan). The expression of anti-TNFα antibody was detected by ELISA, and the positive clones were further screened for high binding affinity.

### Construction of expression vector

We isolated anti-TNFα-positive B cells from the peripheral blood of patients with active RA. The total RNA from the cloned anti-TNFα-positive cells was extracted using Trizol (9109, Takara, Otsu, Shiga, Japan), and cDNA was obtained by Moloney Murine Leukemia Virus (MMLV) reverse transcription (M1701, Promega, Wisconsin, Madison, USA).

**Figure 1.**
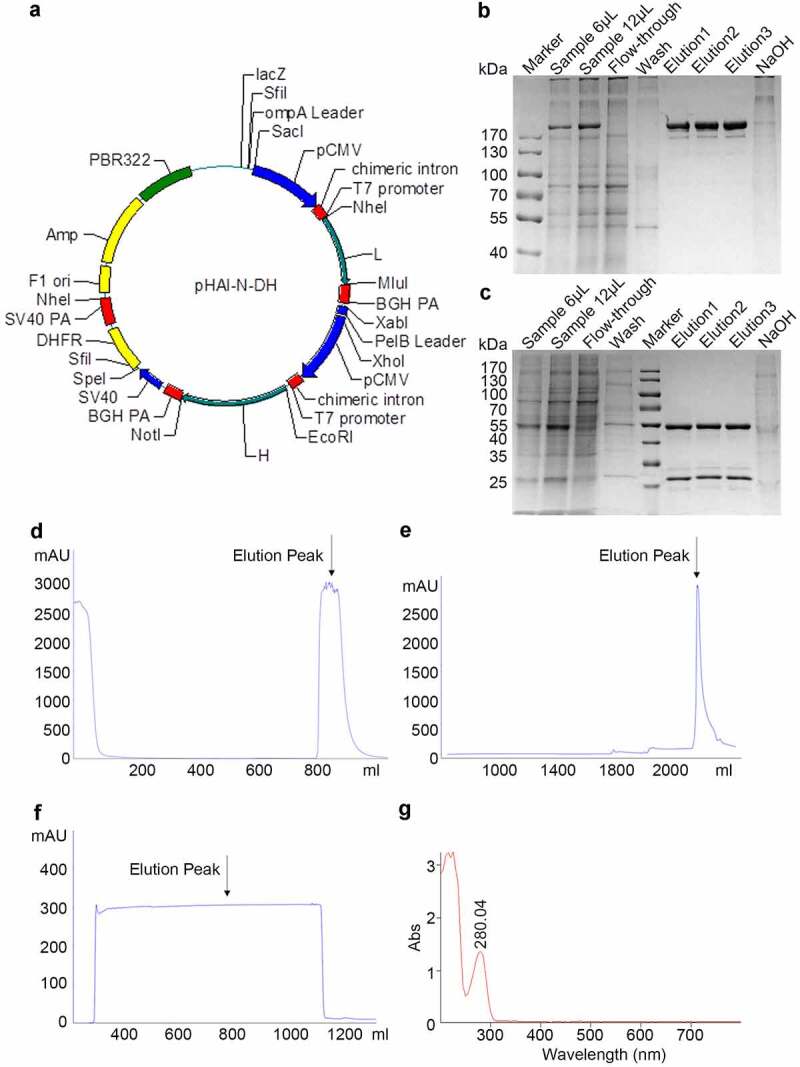
Expression and purification of Haidalimumab. (a) map of expression vector. (b) non-reducing SDS-PAGE electrophoresis illustrated antibodies before and after purification, (c) reducing SDS-PAGE electrophoresis illustrated antibodies before and after purification. Description of wells is as follows: Sample is the supernatant from fermentation, flow-through is the solution that flows out after loading sample and before washing, elution1, elution2 and elution3 are the eluents of the target protein collected at different time points, (d) cell culture supernatant was purified using MabSelect SuRe™ (protein A affinity chromatography), and the elution peak that contained antibodies was identified (the arrow in Figure 1(d)). (e) the eluate from D was then purified by cation-exchange chromatography and the eluate was collected (the arrow in Figure 1(e)). (f) the eluate from E was then purified using anion-exchange chromatography; the eluate (purified Haidalimumab) was collected. (g) the eluate from F showed a specific absorption peak at 280 nm, confirming that Haidalimumab is a protein

The subsequent gene cloning steps, including PCR, sequencing, and expression vector construction, were all performed according to our previous work [24]. The primers used for PCR were as follows: light chain forward primer: 5ʹ-GAAATTGTGCTCACACAGTC-3ʹ, light chain reverse primer: 5ʹ-CTAACACTCTCCCCTGTTGAAGC-3ʹ, heavy chain forward primer: 5ʹ-GAAGTCCAGCTGGTCGAGAG-3ʹ, heavy chain reverse primer: 5ʹ-GTGAGTTTTGTCACAAGATTTGGGCTC-3ʹ. The cDNA sequences of the heavy and light chains of TNF antibody were inserted into plasmid pHAI by double enzyme digestion separately (Light chain restriction sites: Nhe/BamHI, Heavy chain restriction sites: EcoRI/NotI, Takara company). Vector pHAI is an expression plasmid designed and constructed by us. It contains a heavy constant chain and a light constant chain in two reading frames (Figure 1(a)) and carries a neomycin resistance marker for the selection of stable transfectants.

### Transfection of expression vector into CHO cells and screening of cells

CHO cells were maintained at 37°C (5% CO_2_) in Dulbecco’s Modified Eagle’s Medium (SH30022.01B, HyClone) supplemented with 10% (v/v) fetal bovine serum, 100 U/mL penicillin, and 1 mg/mL streptomycin. The cell culture products and reagents were purchased from GIBCO. The expression vector was transfected into CHO cells using the liposome method (Lipofectamine 2000, Invitrogen) [25]. After transfection, 2–300 nM methotrexate (MTX, A7019, Sigma, St. Louis, Missouri, USA) was gradually added to screen the stable cell lines for 3 months.

### Separation and purification of Haidalimumab

Stable cell lines with high antibody expression levels were obtained through methotrexate pressurization. The cell line with the highest antibody expression level was cultured in a 5-L bioreactor (Z310110010, Applikon, Delft, Netherlands). The cell culture supernatant was concentrated and purified using a purification system (AKTA Explorer 10, General Electric Company, Boston, Massachusetts, USA) and a three-step chromatographic method [26] as follows: MabSelect SuRe™ (17,543,802, General Electric Company) affinity chromatography to bind antibodies in the cell culture supernatant; Capto™ S (17,544,101, General Electric Company) cation exchange chromatography to exchange target antibodies by conferring a positive charge according to the isoelectric point of antibody; and Capto™ Q (17,531,602, General Electric Company) anion exchange chromatography to remove endotoxins and nucleic acids from the antibodies.

### Molecular weight and purity of Haidalimumab

The molecular weight and the purity of Haidalimumab obtained from the purification process were determined using SDS-PAGE (sodium dodecyl sulfate polyacrylamide gel electrophoresis) [27]. ExpressPlus™ PAGE Gel and running buffer were purchased from Genscript Biotech Corporation (M42015C, M00138, Nanjing, China). Reducing and non-reducing loading buffers were purchased from Applygen Technologies Inc.(B1033, B1012, Beijing, China). The purity was also analyzed by HPLC (high-performance liquid chromatography), using a 2695 instrument (Waters) and a Phenomenex Biosep SEC S3000 column. The sample loading buffer was made from 0.05 M PBS at pH 6.8 and the cleaning buffer, from 0.1 M Monosodium phosphate (NaH_2_PO_4_) at pH 3.0 (0.22 μm precision filtration, ultrasonic degassing for 20 min). The sample (200 μL) was added into the sample tube ensuring the absence of bubbles in the sample tube and was run for 40 min. The detection wavelengths were 215 nm and 280 nm, and the sample loading flow rate was 0.5 mL/min.

### Protein sequencing

To determine whether the DNA sequence was correctly translated into the expected protein sequence, amino acid sequencing was performed [28]. For N-terminal sequencing, the standard method (SCI-S-006) was followed using ABI PROCISE™ 492cLC sequencer (GC320078, Applied Biosystems, Foster, California, USA). For C-terminal sequencing, a mass spectrometry method was followed using an ABI4800plus MALDI-TOF-TOF analyzer (Applied Biosystems, Foster, California, USA).

### Protein isoelectric point (pI) determination

The isoelectric point of Haidalimumab was determined using the Multiphor II system (General Electric Company) following protocols reported previously [29].

### Binding affinity determination

The binding affinity of Haidalimumab to TNFα was tested using a molecular interactor ForteBio Octet QKe (Pall, New York, USA) [30]. Haidalimumab or Humira (Abbott, Chicago, Illinois, USA) samples were fixed to the protein A sensor, and the deposition curves with different concentrations of the analyte (TNFα protein) were analyzed and combined. The KD (affinity constant) values of the samples were determined. R^2^ should be >0.9. The loading concentration of Haidalimumab or Humira was 2 μg/mL, association concentration of TNF-α protein was 1 μg/mL (58.8 nM).

### Neutralizing activity of Haidalimumab in L929 cells

L929 cells (2 × 10^4^ per well, 100 μL) were plated into a 96-well plate overnight [31]. The antibody solution was prepared by mixing different concentrations of the antibodies with recombinant tumor necrosis factor α (rhTNFα) at 1 ng/mL and actinomycin D (A4262, Sigma) at 2.5 μg/mL. The concentrations of the antibodies were 320, 160, 80, 40, 20, 10, 5, 2.5, 1.25, and 0 ng/mL. A serum-free medium (Gibco CD OptiCHO, A1122203, Thermofisher Scientific) was used as the diluent. The antibody solution (100 μL) was added into each well and the cells were cultured overnight at 37°C (5% CO_2_) in an incubator, after which 20 μL of 5 mg/mL methylthiazolyldiphenyl-tetrazolium bromide (MTT, m2128, Sigma) solution was added into each well. The results were read at 570 nm and 630 nm using a Thermo Fisher Multiskan FC microplate reader.

### Haidalimumab treatment of rhTNFα-induced death in C57BL/6 mice

Female C57BL/6 mice (16–18 g) were provided by Shanghai Jiesijie Laboratory Animal Co. Ltd. (China). The animal experiments complied with the requirements of the Institutional Animal Care and Use Committee of Shanghai Jiao Tong University. The procedure was performed as described previously [32]. C57BL/6 mice were injected intraperitoneally with a mixture of D galactosamine (G0500, Sigma) mixture with rhTNFα (C008, Shanghai Sinobio). Haidalimumab was injected simultaneously at a dose of 0.25, 0.5, or 1.0 mg/kg (Table 2). The mouse number of each group is 10. The mouse survival rate after 24 h was recorded. The mice were then sacrificed, the liver tissues were fixed in formalin, dehydrated by gradient alcohol, and embedded in paraffin; liver tissue necrosis was analyzed by hematoxylin-eosin (HE) staining. The experiments were performed in triplicate and the data were statistically analyzed using Graphpad Prism8 software.

**Table 2.** Group design for Haidalimumab treatment of rhTNFα-induced liver damage in C57BL/6 mice

Group	Group name	Group program D-galactosamine+ rhTNFα+	Animal number (n)
1	Blank control	PBS	10
2	Negative control	Human IgG 2.0 mg/kg	10
3	Positive control	Humira 0.5 mg/kg	10
4	Low-dose	Haidalimumab 0.25 mg/kg	10
5	Medium-dose	Haidalimumab 0.5 mg/kg	10
6	High-dose	Haidalimumab 1.0 mg/kg	10

### Haidalimumab treatment of TNF-Tg mice

The animal experiments complied with the requirements of the Institutional Animal Care and Use Committee of New York University. Previous studies have described a TNF-Tg murine arthritis model (TNF- transgenic mice overexpressing tumor necrosis factor develop chronic inflammatory features, including arthritis and axial inflammation) [33,34]. We administered TNF-Tg mice with Haidalimumab to study arthritis remission and to evaluate the pharmacodynamics of Haidalimumab. TNF-Tg mice (Taconic Biosciences, USA) with mild (clinical score 4–6) or severe (clinical score ≥10) arthritis were intraperitoneally injected with PBS (n = 5) or 0.5 mg/kg body weight Humira (n = 5) or Haidalimumab (n = 5) twice every week for 6 weeks. Each paw was evaluated and scored individually using a 0-to-4 scoring system as follows: paw score of 0, no signs; 1, mild swelling confined to the tarsals or ankle joint; 2, mild swelling extending from the ankle to the tarsals; 3, moderate swelling extending from the ankle to metatarsal joints; and 4, severe swelling encompassing the ankle, foot, and digits, or ankylosis of the limb. The paw scores were summed to yield individual scores (maximum score of 16) for each mouse.

### Inhibition of rabbit fever induced by rhTNFα

The animal experiments complied with the requirements of the Institutional Animal Care and Use Committee of Shanghai Jiao Tong University. Human IgG, Humira, and Haidalimumab were injected into the ear vein of New Zealand White female rabbits (Shanghai Jiesijie Laboratory Animal Co. Ltd.). After 15 min, 5 mg/kg rhTNFα was injected intravenously [35]. The rectal temperature was measured with a thermometer after 4 h. The inhibition rate was defined as follows: Inhibition rate (Table 3) = (increased temperature in the rhTNFα group-increased temperature in the rhTNFα & antibody group)/increased temperature in the rhTNFα group.

**Table 3.** Inhibitory effect of Haidalimumab and Humira on induction of rabbit fever by rhTNFα

Group	Temperature increase (°C)				
Haidalimumab (µg/kg)	rhTNFα	rhTNFα + Haidalimumab	Inhibition rate (%)	rhTNFα + Humira	Inhibition rate (%)
28	0.75 ± 0.05	0.50 ± 0.05	33.30	0.5 ± 0.05	32.00
56	0.75 ± 0.05	0.20 ± 0.05	73.30	0.3 ± 0.05	60.00
112	0.75 ± 0.05	0.00	100.00	0.00	100.00
448	0.75 ± 0.05	0.00	100.00	0.00	100.00

### Statistical analysis

Statistical analyses were performed using GraphPad Prism version 8.3.0 (GraphPad Software, Inc., San Diego, CA, USA). All experiments were independently performed in triplicate at least thrice. P values less than 0.05 were regarded as statistically significant (*P < 0.05, **P < 0.01, ***P < 0.001). The results are shown as the mean± standard deviation (SD).

### Ethics statement

This study was performed in accordance with The Code of Ethics of the World Medical 112 Association (Declaration of Helsinki) and was approved by the ethics committee of Shanghai Jiao Tong University. The animal experiments complied with the requirements of the Institutional Animal Care and Use Committee of Shanghai Jiao Tong University and the requirements of the Institutional Animal Care and Use Committee of New York University respectively.

## Results

In order to develop TNFα antibodies with low immunogenicity and with functions similar to that of human antibodies, we screened and isolated anti-TNFα-positive B cells from patients with rheumatoid arthritis. The respective heavy-chain and light-chain sequences were cloned and were used to generate a stable CHO cell line that could produce the antibody which was named Haidalimumab. Haidalimumab showed a binding affinity comparable to that of the antibody Humira, which is the best TNF inhibitor available on the market. Furthermore, Haidalimumab could effectively neutralize rhTNFα toxicity in a C57BL/6 mouse model, and displayed significant therapeutic effect in a TNF-Tg mouse arthritis model. These results demonstrated that we have developed a high-affinity, fully human anti-TNFα antibody with low immunogenicity and potential therapeutic applications in rheumatoid arthritis or in other autoimmune diseases. A summary of the characteristics of the FDA-approved TNF inhibitors and Haidalimumab is shown in supplementary material Table 1.

### Construction of the expression vector and expression and purification of Haidalimumab

We isolated the anti-TNFα-positive B cells from the peripheral blood of patients with active RA and cloned the DNA sequence of the anti-TNFα antibody. After obtaining the gene sequences (Supplemental Figure 1) through sequencing, we constructed an expression vector carrying Haidalimumab gene (Figure 1(a)).

The expression vector was transfected into CHO cells which then secreted Haidalimumab into the culture medium. The protein sequence of Haidalimumab is shown in Supplemental Figure 2. The CHO cell culture supernatant was purified using MabSelect SuRe™ (Figure 1(d)), which can bind different types of antibodies in the cell culture supernatant. Capto™ S (Figure 1(e)), a cation exchange method, was then used, followed by Capto™ Q (Figure 1(f)), which can remove endotoxins and nucleic acids from antibodies by anion exchange chromatography. Non-reducing SDS-PAGE electrophoresis illustrated antibodies before and after purification in Figure 1(b), reducing SDS-PAGE electrophoresis illustrated antibodies before and after purification in Figure 1(c). Haidalimumab was shown to have an absorption peak at 280 nm, as expected for a protein (Figure 1(g)). The molecular weight of the purified Haidalimumab obtained by non-reducing SDS-PAGE was about 180 kDa. The molecular weight of the heavy chain was determined to be about 55 kDa and the light chain, about 28 kDa through reducing SDS-PAGE. Almost all the target proteins were captured in the fermentation broth, with a low quantity of target protein washed away during the alkaline washing process.

### Purity and isoelectric point determination

**Figure 2.**
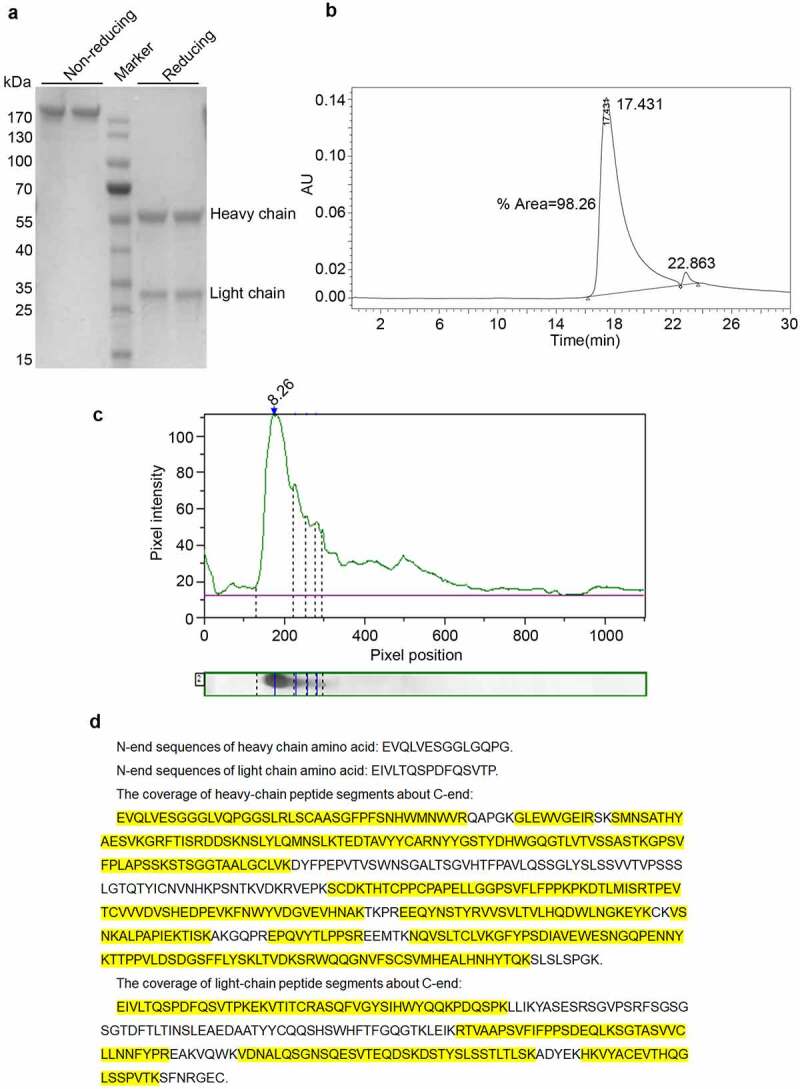
Purity, isoelectric point, and protein sequence analysis of Haidalimumab. (a) reducing SDS-PAGE showed the heavy- and light-chains of the antibody with molecular weights of about 55 and 28 kDa, respectively. Non-reducing SDS-PAGE showed one band at about 180 kDa. (b) the purity of Haidalimumab determined by high-performance liquid chromatography was 98.26%. (c) the isoelectric point of Haidalimumab was about 8.26. (d) protein sequence analysis of Haidalimumab. Amino acid sequences of the N-terminal end of the heavy-chain and the light-chain were EVQLVESGGLGQPG and EIVLTQSPDFQSVTP respectively, which were also consistent with the expected sequence. The coverage of heavy-chain peptide segments toward the C-terminal end had a total of 450 amino acids, 347 amino acids (yellow highlight) were identified, accounting for 77.11% of the total amino acid sequence. The light-chain had a total of 214 amino acids, of which 133 (yellow highlight) were identified, accounting for 62.15% of the total amino acid sequence

Reducing SDS-PAGE showed that Haidalimumab contained two protein components with different molecular weights (Figure 2(a)) corresponding to the heavy and the light antibody chains of molecular weights of approximately 55 and 28 kDa, respectively. Non-reducing SDS-PAGE showed one band at about 180 kDa (Figure 2(a)), consistent with the presence of two heavy chains and two light chains per antibody molecule. Importantly, no other bands were observed (Figure 2(a)), which suggested that the purity of Haidalimumab was >95%. By high-performance liquid chromatography, we determined that the purity of Haidalimumab (Figure 2(b)) was 98.26%; this result exceeded the standard of >95% required by the Chinese Pharmacopoeia. The isoelectric point of Haidalimumab was determined to be 8.26 (Figure 2(c)).

### Protein sequencing

**Figure 3.**
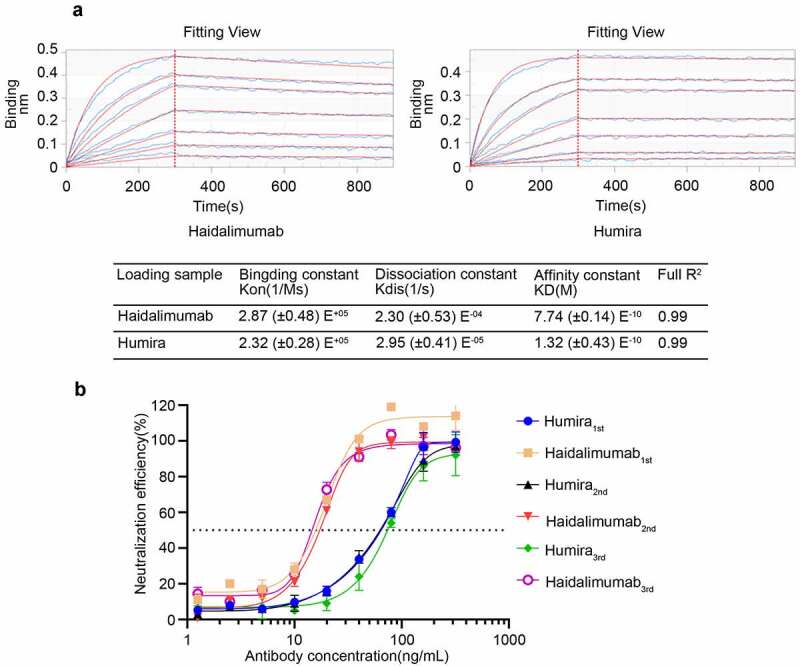
Potency analysis *in vitro.* (a) affinity of Haidalimumab and Humira. The KD (affinity constant) of Haidalimumab for TNFα was 7.74 (± 0.14) E^−^[10]. The KD of Humira was 1.32 (± 0.43) E^−^[10]. (b) neutralizing activity of Haidalimumab and Humira in L929 cells, as calculated from the graphs. The EC50 value of Haidalimumab was 19.498, 17.989, 15.776 ng/mL, while that of Humira was 68.234, 65.163, 74.131 ng/mL (P < 0.01). Haidalimumab_1st_, Haidalimumab_2nd_, and Haidalimumab_3rd_ represent antibodies produced in different batches

To determine whether the DNA sequence was correctly transcribed and then translated into a protein sequence, amino acid sequencing of the N-terminal end of the heavy-chain was performed (Figure 3): The N-terminal sequence of heavy-chain amino acid was EVQLVESGGLGQPG, consistent with the expected sequence. The N-terminal sequence EIVLTQSPDFQSVTP of the light-chain amino acid (Figure 2(d)) was also consistent with the expected sequence. The sequence of the heavy-chain peptides along the C-terminal end, as determined by mass spectrometry, is shown in Figure 2(d). The sample has 450 amino acids totally; 347 amino acids (yellow highlight) were identified by mass spectrometry fragmentation, accounting for 77.11% of the total amino acid sequence. The sequences determined were consistent with the theoretical protein sequence. The light-chain has 214 amino acids totally (Figure 2(d)). Of these, 133 (yellow highlight) were identified by mass spectrometry, accounting for 62.15% of the total amino acid sequence. The sequences determined were consistent with the theoretical sequence of the protein.

### Affinity

The deposition curves with different concentrations of the TNFα protein were analyzed and combined (Figure 3(a)). KD is the affinity constant and the smaller its value, the stronger the affinity of the antibody. As shown in Figure 3(a), the KD of Haidalimumab for TNFα was 7.74 (± 0.14) E^−^[10], while that of Humira was 1.32 (± 0.43) E^−^[10]. Moreover, Haidalimumab presented a significant specificity for rhTNFα. The measured affinity of Humira corresponded with the previously reported value [22,36], confirming the validity of our results.

### The neutralizing activity of Haidalimumab in L929 cells

Haidalimumab could neutralize L929 cytotoxicity (Figure 3(b)). We determined the EC50 (half-effective concentration) values of the antibodies produced in different batches. The EC50 values of Haidalimumab were 19.498, 17.989, 15.776 ng/mL, while that of Humira were 68.234, 65.163, 74.131 ng/mL. This implied that Haidalimumab had a better neutralization effect than Humira. Statistical data analysis showed that there were significant differences between Humira and Haidalimumab in their neutralizing activities in L929 cells (P < 0.01).

### Haidalimumab effect on rhTNFα-induced liver damage in C57BL/6 mice

A subcutaneous injection of rhTNFα in combination with D-galactosamine in C57BL/6 mice leads to acute liver damage [37,38], which manifests with apoptosis and necrosis of a large number of liver cells, significant liver swelling, and congestion. We tested whether Haidalimumab could alleviate liver damage in mice. Mice showed significant liver damage at 800 mg/kg D-galactosamine mixed with 50 μg/kg rhTNFα, and almost all these mice died. We repeated the experiment and obtained similar results, thus establishing 800 mg/kg D galactosamine mixed with 50 μg/kg rhTNFα as the drug concentration for our acute liver injury model. The effect of Haidalimumab was then evaluated. The experimental groups were designed as follows (Table 2).

**Figure 4.**
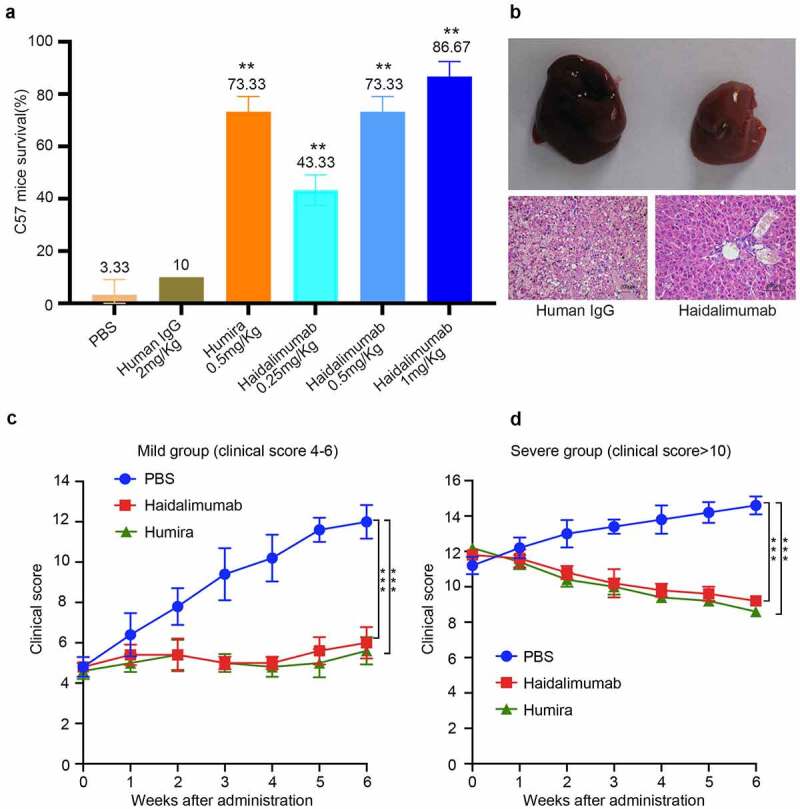
Safety and potency analysis *in vivo.* (a) the protective effect of antibodies on D-galactosamine and rhTNFα-induced liver damage. Survival rate of C57BL/6 mice was calculated after the mice were injected with antibodies for protection from liver damage induced by D-galactosamine and rhTNFα. Haidalimumab at 0.5 mg/kg and 1.0 mg/kg treatment groups showed 73.3% and 86.67% survival rate, respectively, and the survival rate of the Haidalimumab at 0.5 mg/kg treatment group was the same as that of the Humira treatment group. Compared with human IgG group *0.001 < P < 0.05, ** P < 0.001. (b) morphological analysis and hematoxylin and eosin staining analysis of liver damage in mice; the livers of mice in the human IgG-treatment group showed acute damage, manifested as a large number of cells showing apoptosis and necrosis, significant liver swelling, and congestion. In contrast, the livers of Haidalimumab-treated mice had normal morphology of liver tissue. (c) relief from arthritis in TNF-Tg mice with mild (clinical score 4–6, n = 5 mice per group) arthritis were treated; after about a week, the Humira and Haidalimumab treatments were effective and equivalent. (d) relief from arthritis in TNF-Tg mice with severe (clinical score ≥10, n = 5 mice per group) arthritis; after about a week, the Humira and Haidalimumab treatments were effective and equivalent. ***P < 0.001

Haidalimumab of different concentrations was mixed with 800 mg/kg D-galactosamine and 50 μg/kg rhTNFα, and the mixture was injected subcutaneously. The survival rate of each group was calculated 24 h after injection (Figure 4(a)) and was observed to be 3.3% in the phosphate-buffered saline (PBS; negative control) group, 10% in the human IgG at 2.0 mg/kg group, 43.3% in the Haidalimumab at 0.25 mg/kg treatment group, 73.3% in the Haidalimumab at 0.5 mg/kg treatment group, and 86.67% in the Haidalimumab at 1.0 mg/kg treatment group. The survival rate in the three antibody therapy groups was higher than that of the control group (P < 0.05), and the survival rate in the Haidalimumab at 0.5 mg/kg treatment group was the same as that in the Humira at 0.5 mg/kg group. Thus, it was shown that the affinity and the biological activity of Haidalimumab were comparable to those of Humira.

We dissected the mice and found that there were significant differences in the liver morphology between the Haidalimumab-treated and the human IgG-treated groups (Figure 4(b)). Significant liver swelling and congestion were found in the human IgG group, and HE staining analysis revealed that the liver in the human IgG group had a large number of apoptotic and necrotic cells. However, the livers were relatively undamaged in the Haidalimumab group.

### Haidalimumab treatment of TNF-Tg mice

After six weeks of continuous treatment (Figure 4(c)), mice with mild arthritis had a clinical score of 6.0 ± 0.77, 5.6 ± 0.68, and 12.0 ± 0.84 in the Haidalimumab-, Humira-, and PBS-treatment groups, respectively. Thus, arthritis symptoms were significantly improved in the mice with mild arthritis treated with Haidalimumab or Humira compared to the PBS-treated (negative control) group. Meanwhile, after 6 weeks of continuous treatment (Figure 4(d)), mice with severe arthritis had a clinical score of 9.2 ± 0.2 in the Haidalimumab group, and the clinical scores were 8.6 ± 0.24 and 14.6 ± 0.51 in the Humira and PBS groups, respectively. Thus, arthritis symptoms were significantly improved in Haidalimumab-or Humira-treated mice with severe arthritis compared with those in the PBS group. These results showed that Haidalimumab and Humira showed a similar remission effect on a murine arthritis model.

### Inhibition of rabbit fever induced by rhTNFα

The induction of rabbit fever by rhTNFα has been reported previously [35,39]. Haidalimumab could suppress rabbit fever (Table 3). When the dose of Haidalimumab was 112 mg/kg, rabbit fever was completely inhibited. At 28 mg/kg, Haidalimumab had an inhibition rate of 49.33%, whereas the Humira inhibition rate at the same dose was 32%. At 56 mg/kg, the Haidalimumab inhibition rate was 77.33%, and that of Humira was 60%. Thus, Haidalimumab was significantly more efficient at inhibiting rabbit fever than Humira.

## Discussion

Plasma from rehabilitated patients can be used to treat diseases, as it contains neutralizing antibodies. However, this treatment has disadvantages, including limited plasma source; difference in antibody concentrations, activities, and effects of the plasma source; and biosecurity risks. To help resolve these limitations, we generated Haidalimumab, a novel anti-human TNFα monoclonal antibody that has promising clinical application potential. Gene sequences were obtained from the B cells, which showed high levels of secretion of anti-human TNFα, of RA patients. Given that the sequences were derived entirely from humans, a low immunogenicity of the antibody was ensured. A CHO cell line stably expressing anti-human TNFα antibody was constructed using molecular biology techniques. The affinity and the pharmacodynamic characteristics of Haidalimumab assessed *in vitro* and *in vivo* were shown to be comparable to, or even better than, those of Humira.

N-terminal protein sequencing was consistent with the theoretical (expected) sequences of the light and heavy chains of the antibody. In C-terminal sequencing, the coverage of the heavy-chain peptides and the light-chain peptides accounted for 77.11% and 62.15% of the total amino acid sequences, respectively. Even when the detection conditions were adjusted, the coverage did not reach 100%. The reason for this could be protein modifications (e.g. post-translational modifications), N-end closure, or the limitations in the currently existing protein detection technology. Combining methods could presumably overcome this limitation in the future [40]. Subsequent analysis results showed that the activity and the efficacy of Haidalimumab were as good as those of Humira. The specific affinity of Haidalimumab for human TNFα was similar to that of Humira, and the measured affinity of Humira was close to the value reported previously [22,36].

To perform pharmacology experiments in TNF-Tg mice, we used mice with similar characteristics as the Tg-197 mice (hTNF transgenic mice), of which human TNFα overexpression is the primary characteristic. Wei Tang et al. used TNF-Tg mice to evaluate the effect of the growth factor progranulin (PGRN) and revealed that PGRN prevented inflammation in multiple arthritis mouse models and inhibited TNFα-activated intracellular signaling[33]. Shealy et al. used TNF-Tg mice to characterize golimumab, which is a human monoclonal antibody specific for human TNFα[17]. In our TNF-Tg mouse arthritis model, we found that the remission of arthritis due to Haidalimumab treatment was comparable to that of Humira in both moderate arthritis model and or severe arthritis model.

## Conclusion

In summary, we have cloned and isolated a fully-human anti-rhTNFα monoclonal antibody named Haidalimumab in this study. The gene sequences encoding the antibody are of human origin, allowing the antibody to retain its natural properties, particularly low immunogenicity. The affinity of Haidalimumab is comparable to that of Humira, and Haidalimumab proved to be effective in both cell level and animal models. Humira has been approved for more than ten indications, and various studies on this drug have been reported [41,42]. We believe that our drug, Haidalimumab, has excellent clinical application prospects. It is now crucial to speed up the pace of research and development of Haidalimumab so that it can start with being used in clinical applications as soon as possible.

## Supplementary Material

Supplemental Material

## Research highlights

- A fully human monoclonal antibody (Haidalimumab) specific for TNFα was developed.
- Haidalimumab is comparable to Humira in potency and efficacy.
- Haidalimumab has therapeutic potential in RA treatment.
