# Supplementary material for: Preparation and characterization of a fully human monoclonal antibody specific for human tumor necrosis factor alpha
Source: Bioengineered. 2021 Dec 9;12(2):10821–34. doi: 10.1080/21655979.2021.1967710 (PMC8809985; doi:10.1080/21655979.2021.1967710)
Supplement: Supplemental Material [file KBIE_A_1967710_SM3541.zip › supplemental materialsclean.docx]

Supplemental figure 1 Haidalimumab nucleotide sequences

Haidalimumab light chain nucleotide sequences

gaaattgtgc tcacacagtc accagacttt cagtctgtca cccctaagga gaaagtgacc 60

atcacttgca gggcctctca gttcgtcggc tatagtatcc actggtacca gcagaaaccc 120

gatcagtccc ctaaactgct gatcaagtac gcctctgaat caaggtcagg tgtccccagt 180

cgattttctg gatcaggatc tggtaccgac ttcaccctca ccatcaatag cttggaggcc 240

gaggacgctg ctacctacta ctgccaacaa agccacagct ggcactttac ttttggccag 300

gggaccaagc ttgagatcaa acgaactgtg gctgcaccat ctgtcttcat cttcccgcca 360

tctgatgagc agttgaaatc tggaactgcc tctgttgtgt gcctgctgaa taacttctat 420

cccagagagg ccaaagtaca gtggaaggtg gataacgccc tccaatcggg taactcccag 480

gagagtgtca cagagcagga cagcaaggac agcacctaca gcctcagcag caccctgacg 540

ctgagcaaag cagactacga gaaacacaaa gtctacgcct gcgaagtcac ccatcagggc 600

ctgagctcgc ccgtcacaaa gagcttcaac aggggagagt gttag 645

Haidalimumab heavy chain nucleotide sequences

gaagtccagc tggtcgagag cggtggcggg ctggtgcaac ccggtggatc actgcggctc 60

agctgcgctg ctagtggctt tcccttctct aaccactgga tgaattgggt ccggcaggct 120

ccaggaaagg gtctggagtg ggtgggtgag atcaggagta agtctatgaa ctccgccaca 180

cactatgctg aaagcgtgaa agggcgcttc acaatctcta gagacgattc aaagaactct 240

ctgtacctgc agatgaacag tctgaaaaca gaggacaccg ctgtgtatta ctgtgctcgg 300

aactactacg gttcaactta cgaccactgg ggccaaggta cactggtcac cgtctcgagt 360

gcctccacca agggcccatc ggtcttcccc ctggcaccct cctccaagag cacctctggg 420

ggcacagcgg ccctgggctg cctggtcaag gactacttcc ccgaaccggt gacggtgtcg 480

tggaactcag gcgccctgac cagcggcgtg cacaccttcc cggctgtcct acagtcctca 540

ggactctact ccctcagcag cgtggtgacc gtgccctcca gcagcttggg cacccagacc 600

tacatctgca acgtgaatca caagcccagc aacaccaagg tggacaagag agttgagccc 660

aaatcttgtg acaaaactca cacatgccca ccgtgcccag cacctgaact cctgggggga 720

ccgtcagtct tcctcttccc cccaaaaccc aaggacaccc tcatgatctc ccggacccct 780

gaggtcacat gcgtggtggt ggacgtgagc cacgaagacc ctgaggtcaa gttcaactgg 840

tacgtggacg gcgtggaggt gcataatgcc aagacaaagc cgcgggagga gcagtacaac 900

agcacgtacc gtgtggtcag cgtcctcacc gtcctgcacc aggactggct gaatggcaag 960

gagtacaagt gcaaggtctc caacaaagcc ctcccagccc ccatcgagaa aaccatctcc 1020

aaagccaaag ggcagccccg agaaccacag gtgtacaccc tgcccccatc ccgggaggag 1080

atgaccaaga accaggtcag cctgacctgc ctggtcaaag gcttctatcc cagcgacatc 1140

gccgtggagt gggagagcaa tgggcagccg gagaacaact acaagaccac gcctcccgtg 1200

ctggactccg acggctcctt cttcctctat agcaagctca ccgtggacaa gagcaggtgg 1260

cagcagggga acgtcttctc atgctccgtg atgcatgagg ccctgcacaa ccactacacg 1320

cagaagagcc tctccctgtc tccgggtaaa tga 1353

Supplemental figure 2 Haidalimumab amino acid sequences

Haidalimumab light chain amino acid sequences

Glu Ile Val Leu Thr Gln Ser Pro Asp Phe Gln Ser Val Thr Pro Lys

1 5 10 15

Glu Lys Val Thr Ile Thr Cys Arg Ala Ser Gln Phe Val Gly Tyr Ser

20 25 30

Ile His Trp Tyr Gln Gln Lys Pro Asp Gln Ser Pro Lys Leu Leu Ile

35 40 45

Lys Tyr Ala Ser Glu Ser Arg Ser Gly Val Pro Ser Arg Phe Ser Gly

50 55 60

Ser Gly Ser Gly Thr Asp Phe Thr Leu Thr Ile Asn Ser Leu Glu Ala

65 70 75 80

Glu Asp Ala Ala Thr Tyr Tyr Cys Gln Gln Ser His Ser Trp His Phe

85 90 95

Thr Phe Gly Gln Gly Thr Lys Leu Glu Ile Lys Arg Thr Val Ala Ala

100 105 110

Pro Ser Val Phe Ile Phe Pro Pro Ser Asp Glu Gln Leu Lys Ser Gly

115 120 125

Thr Ala Ser Val Val Cys Leu Leu Asn Asn Phe Tyr Pro Arg Glu Ala

130 135 140

Lys Val Gln Trp Lys Val Asp Asn Ala Leu Gln Ser Gly Asn Ser Gln

145 150 155 160

Glu Ser Val Thr Glu Gln Asp Ser Lys Asp Ser Thr Tyr Ser Leu Ser

165 170 175

Ser Thr Leu Thr Leu Ser Lys Ala Asp Tyr Glu Lys His Lys Val Tyr

180 185 190

Ala Cys Glu Val Thr His Gln Gly Leu Ser Ser Pro Val Thr Lys Ser

195 200 205

Phe Asn Arg Gly Glu Cys

210

Haidalimumab heavy chain amino acid sequences

Glu Val Gln Leu Val Glu Ser Gly Gly Gly Leu Val Gln Pro Gly Gly

1 5 10 15

Ser Leu Arg Leu Ser Cys Ala Ala Ser Gly Phe Pro Phe Ser Asn His

20 25 30

Trp Met Asn Trp Val Arg Gln Ala Pro Gly Lys Gly Leu Glu Trp Val

35 40 45

Gly Glu Ile Arg Ser Lys Ser Met Asn Ser Ala Thr His Tyr Ala Glu

50 55 60

Ser Val Lys Gly Arg Phe Thr Ile Ser Arg Asp Asp Ser Lys Asn Ser

65 70 75 80

Leu Tyr Leu Gln Met Asn Ser Leu Lys Thr Glu Asp Thr Ala Val Tyr

85 90 95

Tyr Cys Ala Arg Asn Tyr Tyr Gly Ser Thr Tyr Asp His Trp Gly Gln

100 105 110

Gly Thr Leu Val Thr Val Ser Ser Ala Ser Thr Lys Gly Pro Ser Val

115 120 125

Phe Pro Leu Ala Pro Ser Ser Lys Ser Thr Ser Gly Gly Thr Ala Ala

130 135 140

Leu Gly Cys Leu Val Lys Asp Tyr Phe Pro Glu Pro Val Thr Val Ser

145 150 155 160

Trp Asn Ser Gly Ala Leu Thr Ser Gly Val His Thr Phe Pro Ala Val

165 170 175

Leu Gln Ser Ser Gly Leu Tyr Ser Leu Ser Ser Val Val Thr Val Pro

180 185 190

Ser Ser Ser Leu Gly Thr Gln Thr Tyr Ile Cys Asn Val Asn His Lys

195 200 205

Pro Ser Asn Thr Lys Val Asp Lys Arg Val Glu Pro Lys Ser Cys Asp

210 215 220

Lys Thr His Thr Cys Pro Pro Cys Pro Ala Pro Glu Leu Leu Gly Gly

225 230 235 240

Pro Ser Val Phe Leu Phe Pro Pro Lys Pro Lys Asp Thr Leu Met Ile

245 250 255

Ser Arg Thr Pro Glu Val Thr Cys Val Val Val Asp Val Ser His Glu

260 265 270

Asp Pro Glu Val Lys Phe Asn Trp Tyr Val Asp Gly Val Glu Val His

275 280 285

Asn Ala Lys Thr Lys Pro Arg Glu Glu Gln Tyr Asn Ser Thr Tyr Arg

290 295 300

Val Val Ser Val Leu Thr Val Leu His Gln Asp Trp Leu Asn Gly Lys

305 310 315 320

Glu Tyr Lys Cys Lys Val Ser Asn Lys Ala Leu Pro Ala Pro Ile Glu

325 330 335

Lys Thr Ile Ser Lys Ala Lys Gly Gln Pro Arg Glu Pro Gln Val Tyr

340 345 350

Thr Leu Pro Pro Ser Arg Glu Glu Met Thr Lys Asn Gln Val Ser Leu

355 360 365

Thr Cys Leu Val Lys Gly Phe Tyr Pro Ser Asp Ile Ala Val Glu Trp

370 375 380

Glu Ser Asn Gly Gln Pro Glu Asn Asn Tyr Lys Thr Thr Pro Pro Val

385 390 395 400

Leu Asp Ser Asp Gly Ser Phe Phe Leu Tyr Ser Lys Leu Thr Val Asp

405 410 415

Lys Ser Arg Trp Gln Gln Gly Asn Val Phe Ser Cys Ser Val Met His

420 425 430

Glu Ala Leu His Asn His Tyr Thr Gln Lys Ser Leu Ser Leu Ser Pro

435 440 445

Gly Lys

450

Supplemental table 1 Antibody quality control

| Test items | | Test standard | Results |
| --- | --- | --- | --- |
| pH | | 6.5-7.5 | 6.78 |
| Molecular weight | | 55/29 KDa ± 10% | 55/28 KDa |
| Purity | SDS-PAGE | Monomer ≥90% | ≥ 95% |
|  | HPLC | ≥ 95% | 98.26% |
| UV absorption spectrum (nm) | | 280 ± 2nm | 280nm |
| Asepsis test | | Negative | Negative |
| Host cell protein content (%) | | ≤ 0.1 | ≤0.05 |
| Bacterial endotoxin (EU/mg.pro) | | ≤ 2.0 EU/mg | ≤2.0 EU/mg |
| Protein A residue (%) | | ≤ 0.1 | ≤0.05 |
| N-terminal amino acid sequence | | Consistent with design | Consistent with design |
| C-terminal amino acid sequence | | Consistent with design | Consistent with design |
| Affinity test | | Affinity constant KD ≈10^-10^ M | 7.74 × 10^-10^ M |
